# 

**Published:** 1887-06-11

**Authors:** 


### The Hospital.

**An Institution, Family, and Parochial Journal of Hospitals, Asylums, and all Agencies for the Care of the Sick, Criticism and News.**

SATURDAY, JUNE 11TH, 1887.

### HOSPITAL SATURDAY.

### THE LITERARY PRIZE.

A PRIZE of **Two Guineas** will be given every month for the best story or incident based upon hospital, or asylum, or student life, or any incident connected with the professions of medicine or nursing.

*** THE CHILDREN'S CORNER and AMUSEMENTS WITH PROFIT will be found on page vi of this week's issue.

### THE IN- AND OUT-PATIENTS' HOSPITAL DIARY.

This Diary is so arranged as to make some portion of it appear every week, and the whole in each monthly part. It has been very difficult to compile, and corrections will at all times be gratefully received. It has been suggested that similar information about the larger Provincial and Scotch Hospitals would be useful to many readers. We should be glad to hear if this is felt to be the case.

### I.—GENERAL HOSPITALS.

| Hospitals and Terms of Admission. | Physicians, Surgeons, and Medical Officers, with Days and Hours of Attendance. |
|---|---|
| CHARING CROSS HOSPITAL, West Strand, W.C. Sec., Mr. A. E. Reade<br>Urgent cases are immediately admitted as in-patients. Subscriber's letter expected in other cases<br>Out-patients admitted without subscriber's letter first time; for continued attendance they must be obtained from the Secretary | *In-Physicians*, Drs. Pollock, M. Th.; Green, W. S.; Bruce, Tu. F. Hour, 1.30<br>*In-Surgeons*, Messrs. Barwell, M. Th.; Bellamy, Tu. F.; Bloxam, W. S. Hour, 2<br>*Out-Physicians*, Drs. Wilcocks, M.Th.; Abercrombie, Tu. F.; Lubbock, W. S.; Murray, W. S. Hour, 1.30<br>*Out-Surgeons*, Messrs. Cantlie, M.Th.; J. H. Morgan, Tu. F.; Stanley Boyd, W. S. Hour, 1.30 |
| "DREADNOUGHT" HOSPITAL (Seamen's Hospital Society), Greenwich, S.E. Sec., Mr. W. T. Evans<br>Entirely free to seamen and to all accident cases | *In- and Out-Physicians*, Drs. Curnow, Tu. Th. S.; H. T. Griffiths, M.W.F. Daily, between 9 and 3<br>*In- and Out-Surgeons*, Messrs. W. J. Smith, G. R. Turner. Daily, between 9 and 3 |
| FRENCH HOSPITAL, 10, Leicester Place, Leicester Sq., W.C. Sec., Mr. E. Rimmel<br>Free to urgent cases and to all foreigners speaking French | *In-Physician*, Dr. Vintras, Tu. S. 10<br>*In-Surgeons*, Sir W. MacCormac, Tu. 9; Mr. J. Késer, W. F. 10<br>*Out-Physician*, Dr. Vintras, Tu. S. 10<br>*Out-Surgeons*, Messrs. H. de Méric, M. Th. 10; J. Késer, W. F. 10 |
| GERMAN HOSPITAL, 113, Dalston Lane, E. Sec., Mr. C. Feldmann<br>Free to natives of Germany, and others speaking German, and to all accidents and emergencies. Others by Governors' letters | *In-Physicians*, Drs. Weber, M. T. 2; Port, Tu. F. 9 a.m.<br>*In-Surgeons*, Drs. Lichtenberg and Burger, W. S. 2<br>*Out-Physicians*, Drs. Tuchmann, Tu. W. 2; Ludwig, M. Th. 2; Keller, Tu. W. F. 2; Philippi, M. Th. F. 2 (*Men*, Tu. Th.; *Women*, M. W. F.) |
| ——— Eastern Branch, 49, Mansell Street, Aldgate, E. | *Out-Physician*, Dr. C. Harrer, Tu. F. 8 to 10 a.m. |
| ——— Western Branch, 259, Oxford Street, W. | *Out-Physician*, Dr. M. Castaneda, M. T. 8 to 10 a.m. |
| GREAT NORTHERN CENTRAL HOSPITAL, Caledonian Road, N. Sec., Mr. W. T. Grant<br>Free to all, without letters of recommendation | *In-Physicians*, Drs. Cholmeley, Cook, and Burnett, M. Tu. W. Th. F. 2<br>*In-Surgeons*, Messrs. W. Adams, W. Spencer Watson, and J. Macready, M. Tu. W. Th. F. 2<br>*Out-Physicians*, Drs. Beale, M. Th. 2; Beevor, Tu. 2, S. 10<br>*Out-Surgeons*, Messrs. Lockwood, M. Th. 2; Makins, Tu. F. 2 |
| GUY'S HOSPITAL, St. Thomas's Street, S.E. Supt., Dr. C. J. Steele<br>Patients are admitted, if beds are available, without letters of recommendation, on application at the Hospital. Out-patients are seen free without letters of recommendation | *In-Physicians*, Drs. Pavy, Tu. F.; Pye-Smith, M. Th. S.; Taylor, M. Tu. Th. F. Hour, 1.30<br>*In-Surgeons*, Messrs. Bryant, M. Th. Durham, M. Th. F.; Howse, W. S.; D. Colley, M. Th. Hour, 1.30<br>*Out-Physicians*, Drs. Carrington, W.; Hale White, M.; Goodhart, M. Tu. Th. F. Hour, 12.30<br>*Out-Surgeons*, Messrs. Lucas, Th.; Golding Bird, S.; Jacobson, W.; Symonds, M. Hour, 12.30 |
| KING'S COLLEGE HOSPITAL, Portugal Street, W.C. Sec., Mr. T. Mosse Macdonald<br>Accidents and urgent cases free at all times. Other cases by Governors' letters, but where not obtainable, application may be made to the Secretary | *In-Physicians*, Drs. Beale, Duffin, Yeo, Tu. W. F. 1.30; Tu. W. F. S. 2<br>*In-Surgeons*, Mr. Wood, Sir J. Lister, Messrs. H. Smith, W. Rose. Daily, 1.30<br>*Out-Physicians*, Drs. Ferrier, J. Curnow, N. I. C. Tirard. Daily, 1.<br>*Out-Surgeons*, Messrs. W. Rose, W. Cheyne, A. B. Barrow. Daily, 1. |
| LONDON HOSPITAL, Whitechapel Road, E. Sec., Mr. A. Haggard<br>Accidents and urgent cases free; out-patients for dental and maternity departments need no recommendation; other cases by subscriber's order. Out-patients are required to attend only once, unless otherwise directed by the Physician or Surgeon | *In-Physicians*, Drs. Mackenzie, Tu. F.; Down, Tu. F.; H. Jackson, M. Th.; Sutton, M. Th.; Fenwick, Tu. F. 1.30<br>*In-Surgeons*, Messrs. Rivington, Tu. F.; Couper, Waren Tay, M. Th.; McCarthy, W.S.; Treves, Tu.F. Hour, 1.30<br>*Out-Physicians*, Drs. Sansom, Th.; M. Ralfe, M. Th.; Turner, Tu. F.; Warner, Tu. F.; Gilbart Smith, W. S. J. Anderson, W. S. Hour, 1.30<br>*Out-Surgeons*, Messrs. Mansell-Moullin, M. Th.; Eve, M. W.; Reeves, Tu. S.; H. Fenwick, Tu.F. Hour, 1.30 |
| LONDON TEMPERANCE HOSPITAL, Hampstead Road, N.W.<br>For treatment without alcohol. By letter or small payment | *In- and Out-Physicians*, Drs. J. Edmunds, M.; J. J. Ridge, Tu. F. Hour, 1.30<br>*In- and Out-Surgeons* Mr. A. P. Gould Th. 2.30 |
| METROPOLITAN FREE HOSPITAL, Kingsland Road, N. Sec., Mr. G. Croxton<br>Admission free to the sick poor, without any letter of recommendation. Urgent cases at all times | *In- and Out-Physicians*, Drs. Drysdale, Tu. F. 12; J. G. Dudley, W. S. 12; Fotherby, M. Th. 12; H. H. Tooth, Tu. F. 9; A. Haig, W. S. 9; A. Davies, M. Th. 9<br>*In- and Out-Surgeons*, Messrs. E. J. Chance, W. S. 9; Goodsall, Tu. F. 9; Walsham, M. Th. 9; A. A. Bowlby, F. 9; S. Paget, Th. 9; C. J. Thompson, daily, 9 |
| MIDDLESEX HOSPITAL, Berners Street, W. Sec. and Supt., Mr. A. O. D. Bartholeyns<br>Urgent cases admitted at all times; other cases by ticket. All medical cases must, in the first instance, be seen by the Resident Medical Officer, who attends at the hospital daily, at 11 a.m. | *In-Physicians*, Drs. Cayley, M. Th.; Coupland, Tu. F.; D. Powell, M.Th.; Finlay, Tu. F. Hour, 1.30<br>*In-Surgeons*, Messrs. Hulke, M. Th.; Lawson, M. Th.; Morris, Tu. F. Hour, 1.30<br>*Out-Physicians*, Drs. Fowler, Tu. F. 3.30; Biss, M. Th. 1.30; Pringle, W. S. 1.30<br>*Out-Surgeons*, Messrs. Andrew Clark, M. 1.15 (*Men*); Th. 1.15 (*Women*); Pearce Gould, F. 1.30 (*Men*); Tu. 1.30 (*Women*); J. B. Sutton, W. S. 9 |
| MILLER HOSPITAL AND KENT DISPENSARY, Greenwich, S.E. Hon. Sec. Mr. W. Bristow. | *Physicians*, Drs. T. Creed, C. H. Hartt, G. Willes<br>*Surgeons*, Messrs. T. Moore, J. Poland, E. Clarke |
| NORTH-WEST LONDON HOSPITAL, 18, Kentish Town Road, N.W. Sec., Mr. A. Craske<br>By subscribers' tickets or payment, but accidents and urgent cases free at all times | *In- and Out-Physicians*, Drs. W. C. Hood, J. Shaw, Edwarde H. Campbell. Daily, 1.30<br>*In- and Out-Surgeons*, Messrs. F. Durham, Collier, Jennings, J. Black. Daily, 1.30 |
| OSPEDALE ITALIANO, 41, Queen Square, Bloomsbury. Sec., Sig. L. Bonacina<br>Free to all, but Italians have preference | *In- and Out-Physicians*, Drs. M. Castaneda, Tu. F. 10 to 11; B. Sassella, M.Th. 10 to 11; Mr. J.W. B. Steggall, W. S. 4 to 5 |
| POPLAR HOSPITAL, 303, East India Dock Road, E. Sec., Lt.-Col. Feneran<br>Free to accidents and emergencies | *In-Surgeons*, Messrs. F. M. Corner, M. Brownfield, and Resident Surgeons<br>*Out-Surgeons*, Dr. J. D. Grant, W. S. Mr. T. E. Bowkett, Tu. F. 12 to 2 |
| ROYAL FREE HOSPITAL, Gray's Inn Road, W.C. Sec., Mr. J. S. Blyth<br>Admission free to the sick poor, without any letter of recommendation | *In- and Out-Physicians*, Drs. Cockle, M. Th.; Sainsbury, Tu. F.; West, W. S. Hour, 1<br>*In- and Out-Surgeons*, Messrs. Rose, M. Th.; W. Gant, Tu. F.; Barrow, W. S. Hour, 1 |
| ST. BARTHOLOMEW'S HOSPITAL, Smithfield. Clerk, Mr. W. Cross<br>Admission free, without letter or ticket. Accidents and other urgent cases admitted at any time; ordinary cases of disease daily, between 9 and 10. Patients are seen for Electrical treatment on M. Tu. Th. and F. 1.30 | *In-Physicians*, Drs. Andrew, Church, Gee, Sir D. Duckworth. Daily, 1.30<br>*In-Surgeons*, Messrs. Savory, T. Smith, Willett, Langton, M. Baker. Daily, 1.30<br>*Out-Physicians*, Drs. Hensley, M. Th. 11; Brunton, Tu. F. 11; Legg, W. S. 11; N. Moore, Tu. W. Th. S. 9<br>*Out-Surgeons*, Messrs. Marsh, Tu. F.; 12; Butlin, M. Th. 12; Walsham, W. S. 12; Cripps and Bruce Clarke, daily, 9<br>*Casualty Physicians*, Drs. Davies, Tu. W. F. S.; O. Browne, M. Tu. Th. F.; Nias, M. W. Th. S. Hour, 9 |
| ST. GEORGE'S HOSPITAL, Hyde Park Corner, S.W. Sec., Mr. C. L. Todd<br>Free to accidents and emergencies. Other in-cases by Governor's or subscriber's letter, but, in necessitous cases, this recommendation is not strictly insisted on. Letters are not required for out-patients. Vaccination on Th. at 11 | *In-Physicians*, Drs. Wadham, M. F.; Dickinson, M. Tu. Th. F.; Whipham, Tu.W. S.; Cavafy, Tu. S. Hour, 1<br>*In-Surgeons*, Messrs. Holmes, M. Th. F. 1, W. 1.30; Rouse, M. Th. F. 1, W. 1.30; Haward, Tu. Th. S. 1, W. [illegible].30<br>*Out-Physicians*, Drs. Ewart, M. F.; Owen, Tu. S. Hours, 10.30 to 12<br>*Out-Surgeons*, Messrs. Bennett, M. F.; Dent, Tu. S. Hours, 10.30 to 12 *Men*, F. S.; *Women*, M. Tu. |

### THE CHILDREN'S CORNER.

**Third Quarterly Prize Competition.**
Began 2nd Apr., 1887 ; ends 25th June, 1887.

**Puzzles—Eleventh Week.**

No. 43. HISTORICAL ENIGMA. My first is a well-known quadruped ; my second is a shallow part of a river ; and my whole an English city which received Charles I after his defeat at the Battle of Naseby.

No. 44. BIBLICAL CHARADE. Five hundred begins it, five hundred ends it, five in the middle, first of all letters, first of all figures. My whole is the name of a King in the Bible.

No. 45.

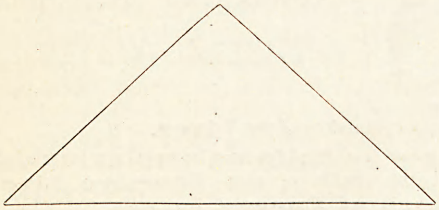

Divide the above figure into four similar and equal parts.

No. 46. I am a word of six letters. My 2, 3, 4 is a breakfast-dish ; my 2, 3, 1, 5 is an envious feeling ; and my 6, 3, 1 is part of a verb.

**Letters—Eleventh Week.**

The Letter-subject for next week will be "Your favourite King or Queen."

**Results of Ninth Week—Puzzles.**

No. 35. CONUNDRUM. Because *you* and *I* ("u" and "i") are in it ; *I'm* ("i," "m") always to be found there ; no omnibus, in fact, is ever without *us* ("u," "s").

No. 36. PUZZLE SENTENCE. "He does not see you because he is without an eye (he s = he *is* without an "i"). Some other clever readings have been received.

No. 37. DROPPED VOWEL PUZZLE. The best physicians are Dr. Diet, Dr. Quiet, and Dr. Merryman.

No. 38. ENIGMA. Cr-ow = Crow.

*All right*—Scrooge, Skye ; *Three right*—A. C. K., Bohemian Girl, Dot, Gom, Joe, A. G. S. McCulloch, Nordisa, Mikado, Tim, Umslopogaas, Pluffy ; *Two right*—Alsie, Ellie, The Eda, Sutton, Violin ; *One right*—R. Epton.

**Letters.**

Our tastes vary, and it is as well that they should, but there are some things which most people enjoy. The arts, for instance, rarely fail to command our admiration. If we cannot ourselves play, we like to listen to beautiful music, and if we cannot draw and paint, still we like to look at lovely pictures. Art—I mean pictorial art—has made marvellous strides during the reign of our good Queen. Our illustrated journals—even our comic papers—show what facile pencils are at work. With those who have a taste for it, drawing is a delightful luxury ; its pursuit excites our admiration of Nature's exquisite handiwork ; it lifts us, as it were, out of ourselves, above the petty vexations of life, to a fuller realisation and a deeper consciousness of the beauty and perfection of everything which God has made.

Some of my young friends have not yet commenced to learn drawing. The most thoughtful letter which I have received this week is decidedly the following from Skye :—

DEAR MR. EDITOR,—I do like drawing : I could paint and draw all day long. I have been working at freehand drawing for a long time at the Art School to which I go, but my drawing-master says that he thinks I have done about enough of *that*, as I have been in for two examinations in it. The *first* (in May last year) I failed in, and I shall not know until August next whether I have passed *this* May or not. I tried to draw Rochester Castle once, but my drawing-master said that I was too ambitious, and that I ought to try some simpler object in drawing from nature, such as a gate or tree, etc. I do not know what the world would be without pictures : the poorest persons can have them now about their houses—they are so cheap. The books long ago must have been very uninteresting without them. I read a very interesting account of Millais' life when he was young (given by himself the other day). He said that when he was about nine years old he competed for a prize in drawing with young men three times his age. When the judge called out little Millais as being the successful competitor, there was no one to be seen, the table being so high, and he such a little boy, that he had to be lifted on to a chair to receive his prize.

I remain, yours faithfully, SKYE.

4, Milton Terrace, Chatham.

One mark each :—Violin, Skye, Mikado, Bohemian Girl, Ellie.

**Notices to Correspondents.**

UMSLOPOGAAS.—The Authorised Version gives "Charity," and this translation is generally accepted. The Greek (ἡ ἀγαπη) can of course be rendered "love," for fundamentally there is little difference.

Answers must be addressed to the "PUZZLE EDITOR," THE HOSPITAL, Norfolk House, Norfolk Street, Strand, W.C., not later than Thursday next. The "Children's Corner" Coupon (see advertisement pages) must be enclosed.

### AMUSEMENTS WITH PROFIT.

### PRIZE COMPETITIONS.

**Quarterly Prize Competition.**
Began 23rd April ; ends 16th July.

**No. 15. Double Acrostic.**

My *initials* and *finals* have both the same sound.
Though widely they vary in rend'ring.
When sad forms of suff'ring my *primals* surround,
Oh ! then may my *finals* attend them.

1. In villages remote from town my form is seen,
With arm uprais'd I stand in stately mien.
2. Backwards and forwards I'm ever the same,
Old-fashioned, perhaps, a fair woman's name.
3. Without thy help the day were darkest night,
Shine forth ! and guide my faltering steps aright !
4. For freedom from a foreign yoke these brave men bled,
And rallied round their Queen when she to combat led.
5. How peaceful is this hour, the day declines,
And from her silver shade the fair moon shines.
6. Hadst thou been good as thou wert brave,
No foreign soil had own'd thy grave.
7. Indolent, happy-go-lucky is he,
'Spite of im-pe-cu-ni-os-i-ty.
8. The spring is here 'neath bough and brake.
A form I spy which sets my heart aquake.

**No. 16. Arithmetical Enigma.**

A hundred and one by fifty divide,
And then to the whole be a cipher applied,
And when this is done, if I rightly divine,
The amount of the sum will be "one" out of "nine."

**Answers.**

No. 11. CHARADE.

The answer is Bass-tin-ado=Bastinado, or Bastinade, as the word is more correctly—but less usually—written.

Correct answers have been received from Ellice, Aralc, Mater, Boss, Tabs, Broadwater, Dolphin, K. M., Gip, Dawnlover, Robin, Letheàdor, A. M. E., Gretta, Ostrich, Perseverance, May, Man of the Sea, Canice, Hexameter, Common Sense.

No. 12. MATHEMATICAL QUESTION.

The long hand passes over twelve minutes while the short hand passes over one.

∴ the hands must first coincide again at 12-11 of 5 minutes past 1 o'clock, and they will continue to overtake each other at a like interval eleven times in the twelve hours.

Ans. 5 and 5-11 minutes past one.

Correct answers have been received from Dawnlover, Letheàdor, Ellice, Canice, Man of the Sea, A. M. E., Perseverance, Ostrich, Hexameter, Gretta, K. M., Dolphin, Broadwater, Boss, Mater.

**The Tie.**

Both Ostrich and Ellice have again correctly guessed the Tie Acrostic as follows :—MartleT ; AgE ; ClaiM ; BishoP ; EyE ; ThersiteS : HuberT.

We now set the following for them :—

"A most beautiful Pagan, a most sweet Jew" ;
In his own conceit, the 'umblest person going :
'Tis a shepherdess small—her name's known to you ;
Alonzo's ghost claim'd this to him owing ;
A fam'd flying isle, Swift's pen brought to view ;
Roundhead and Royalist here fought a drawn game ;
These Furies bore a euphemistic name.

Answers must be addressed to THE PRIZE EDITOR, AMUSEMENTS WITH PROFIT, THE HOSPITAL, Norfolk House, Norfolk Street, Strand, W.C., not later than the *first* post on Friday next. The full name and address must in all cases be given, but a *nom de plume* may be added for publication. Only one side of the paper should be written on. The "Amusements with Profit" Coupon (see advertisement pages) must be enclosed with replies.

